# The rate of 2nd revision for shoulder arthroplasty as analyzed by the Australian Orthopaedic Association National Joint Replacement Registry (AOANJRR)

**DOI:** 10.1080/17453674.2020.1871559

**Published:** 2021-01-12

**Authors:** David R J Gill, Richard S Page, Stephen E Graves, Sophia Rainbird, Alesha Hatton

**Affiliations:** a Orthopaedics Central, Monash Avenue, Nedlands;; b Barwon Centre of Orthopaedic Research and Education, Deakin University, Geelong;; c Australian Orthopaedic Association National Joint Replacement Registry (AOANJRR), Adelaide;; d South Australia Health and Medical Research Institute (SAHMRI), Adelaide, South Australia, Australia

## Abstract

Background and purpose — The increase in shoulder arthroplasty may lead to a burden of revision surgery. This study compared the rate of (2nd) revision following aseptic 1st revision shoulder arthroplasty, considering the type of primary, and the class and type of the revision.

Patients and methods — All aseptic 1st revisions of primary total reverse shoulder arthroplasty (rTSA group) and of primary total stemmed and stemless total shoulder arthroplasty (non-rTSA group) procedures reported to our national registry between April 2004 to December 2018 were included. The rate of 2nd revision was determined using Kaplan–Meier estimates and comparisons were made using Cox proportional hazards models.

Results — There was an increased risk of 2nd revision in the 1st month only for the rTSA group (n = 700) compared with the non-rTSA group (n = 991); hazard ratio (HR) = 4.8 (95% CI 2.2–9). The cumulative percentage of 2nd revisions (CPR) was 24% in the rTSA group and 20% in the non-rTSA group at 8 years. There was an increased risk of 2nd revision for the type (cup vs. head) HR = 2.2 (CI 1.2–4.2), but not class of revision for the rTSA group. Minor (> 3 months) vs. major class revision, and humeral revision vs. all other revision types were second revision risk factors for the non-rTSA group.

Interpretation — The CPR of revision shoulder arthroplasty was > 20% at 8 years and was influenced by the type of primary, the class, and the type of revision. The most common reasons for 2nd revision were instability/dislocation, loosening, and infection.

The volume of shoulder arthroplasty surgery is increasing, and with it the expectation of future revision surgery (Lübbeke et al. 2017, AOANJRR 2019). The survivorship for anatomic shoulder arthroplasty has been well documented (Singh et al. 2011, Page et al. 2014, 2018, Dillon et al. 2019). Previously, revising anatomic shoulder arthroplasty often necessitated bone grafting and re-cementing of the glenoid component. This was associated with soft tissue failure and graft reabsorption (Scalise and Iannotti 2008). Reverse total shoulder arthroplasty (rTSA) offered an opportunity to solve some of these problems (Boileau et al. 2006). Early reports indicated a high complication rate, but satisfactory clinical outcomes (Melis et al. 2012).

The outcome of lower limb revision arthroplasty has demonstrated higher subsequent revision rates compared with known primary procedures (AOANJRR 2018). Similar analysis for shoulder arthroplasty has been more limited, concentrating on specific component revisions or technical descriptions (Cheung et al. 2008, Scalise and Iannotti 2008, Melis et al. 2012, Bonnevialle et al. 2013). These studies have been on small numbers and of short duration. A larger population-based study enables new insights, with a larger number of revision arthroplasty procedures for analysis.

The Australian Orthopaedic Association National Joint Replacement Registry (AOANJRR) has a high enrolment rate. This allows the opportunity to accurately plot population-based shoulder arthroplasty outcomes based on a large volume of implants and across a broad range of diagnoses and surgeons.

This study determined the rate of (second) revision following aseptic first revision shoulder arthroplasty, taking into account the type of primary shoulder arthroplasty revised, and the class of revision undertaken.

## Patients and methods

The AOANJRR began data collection on September 1, 1999 and includes data on almost 100% of the hip and knee arthroplasty procedures performed in Australia since 2002. Data collection was expanded to include shoulder arthroplasty procedures in April 2004 and has documented 97.1% shoulder arthroplasty procedures Australia-wide since November 2007. These data are externally validated against patient-level data provided by all Australian state and territory health departments. A sequential, multilevel matching process is used to identify any missing data, which are subsequently obtained by follow-up with the relevant hospital. Each month, in addition to internal validation and data quality checks, all primary procedures are linked to any subsequent revision involving the same patient, joint, and side. Data are also matched bi-annually to the Australian National Death Index data to identify patients who have died.

In this study, the 1st revisions of primary total reverse shoulder arthroplasty (rTSA group) and total stemmed and stemless (non-rTSA group) procedures performed between April 16, 2004 and December 31, 2018 were analyzed. Resurfacing arthroplasty and hemiarthroplasty were excluded. Revision was defined as removal/exchange or addition of a joint replacement implant. The type of shoulder arthroplasty was the description of the implant (e.g., rTSA or non-rTSA) and the type of revision was the specifically removed/exchanged component (e.g., glenoid articular insert). Revision was further categorized as minor or major. A minor revision involved an exchange of implant not fixed to bone. A major revision involved an exchange of a component with bone fixation on both the glenoid and humeral sides, whilst a partial major revision exchanged bone-fixed components on either the glenoid or humeral side exclusively. A humeral revision included either metaphyseal or both metaphyseal and diaphyseal components. All 1st revisions for infection were excluded to remove the confounding effect on subsequent revisions. The outcome measure was time to 2nd revision for all diagnoses including infection to capture all subsequent revision endpoints.

### Statistics

Kaplan–Meier estimates of survivorship were used to report the time to second revision, with censoring at the time of death and closure of the dataset at the end of December 2018. The unadjusted cumulative percentage revisions (CPRs), with 95% confidence intervals (CI), were calculated using unadjusted point-wise Greenwood estimates. The CPR is displayed until the number at risk for the group reaches 40, unless the initial number for the group is less than 100, in which case the cumulative percentage revision is reported until 10% of the initial number at risk remains. Age- and sex-adjusted hazard ratios (HR) were calculated from Cox proportional hazard models to compare the rate of second revision between groups. The assumption of proportional hazards was checked analytically for each model. If the interaction between the predictor and the log of time was statistically significant in the standard Cox model, then a time-varying model was estimated. Time points were selected based on the greatest change in hazard, weighted by a function of events. Time points were iteratively chosen until the assumption of proportionality was met and HRs were calculated for each selected time-period. For the current study, if no time-period was specified, the HR was calculated over the entire follow-up period. All tests were 2-tailed at 5% levels of significance. Statistical analysis was performed using SAS software version 9.4 (SAS Institute Inc., Cary, NC, USA).

### Ethics, funding, and potential conflicts of interest

The AOANJRR is approved by the Australian Federal Government as a federal quality assurance activity under Section 124X of the Australian Federal Health Insurance Act 1973. All investigations were conducted in accordance with the ethical principles of research (The Helsinki Declaration II). The AOANJRR is funded by the Commonwealth of Australia Department of Health and Ageing. The data of the AOANJRR is the intellectual property of the Australian Orthopaedic Association. The authors declare no financial disclosures.

## Results

There were 1,845 1st revisions of primary total shoulder arthroplasty, with 154 excluded as septic 1st revisions during the study period. Of those remaining, 41% (n = 700) were in the rTSA group, and 59% (n = 991) were in the non-rTSA group. Among the rTSA group 96% (n = 258) remained reverse shoulder arthroplasty at revision and 83% (n = 710) of the non-rTSA group were converted to a reverse shoulder arthroplasty at revision. There were 429 minor revisions, 232 were partial major, and 39 were major in the rTSA group. In the non-rTSA group 128 were minor, 621 were partial major, and 242 were major. Amongst the rTSA group the revisions by type were 270 head/cup, head only, or cup only, 64 glenoid, 166 humeral, and 39 humeral/glenoid. In the non-rTSA group 123 humeral head/glenoid insert or humeral head only, 66 glenoid, 553 humeral, and 242 humeral/glenoid components were revised (Table 1).

**Table 1.** Characteristics of rTSA group and non-rTSA group (all diagnoses, excluding 1st revision for infection)

Variable	Non-rTSA group		rTSA group	
	2nd revisions	1st revisions	2nd revisions	1st revisions
	n = 157 (15.8%)	n = 991	n = 129 (18.4%)	n = 700
	n (% of 1 st rev.)	n (%)	n (% of 1 st rev.)	n (%)
Female sex		594 (59.9)		367 (52.4)
Age				
< 55	14 (18.7)	75 (7.6)	4 (19.0)	21 (3.0)
55–64	57 (20.2)	282 (28.5)	25 (27.2)	92 (13.1)
65–74	61 (15.0)	407 (41.1)	54 (19.4)	279 (39.9)
≥ 75	25 (11.0)	227 (22.9)	46 (14.9)	308 (44.0)
Primary diagnosis^a^				
Fracture	1 (5.9)	17 (1.7)	28 (25.0)	112 (16.9)
Osteoarthritis	142 (15.4)	923 (94.6)	54 (19.2)	281 (42.4)
Osteonecrosis	5 (31.3)	16 (1.6)	0 (0.0)	6 (0.9)
Rheumatoid arthritis	2 (20.0)	10 (1.0)	3 (15.8)	19 (2.9)
Rotator cuff arthropathy	3 (30.0)	10 (1.0)	36 (14.7)	245 (37.0)
Class of 1st revision				
Minor	67 (52.3)	128 (12.9)	81 (18.9)	429 (61.3)
Major partial	56 (9.0)	621 (62.7)	40 (17.2)	232 (33.1)
Major total	34 (14.0)	242 (24.4)	8 (20.5)	39 (5.6)
Type of 1st revision^b^				
Head/insert revision^c^	25 (78.1)	32 (3.3)	0 (0.0)	1 (0.2)
Head only revision^c^	41 (45.1)	91 (9.2)	13 (10.7)	121 (22.4)
Cup only^c^			38 (25.7)	148 (27.5)
Humeral/glenoid revision	34 (14.0)	242 (24.6)	8 (20.5)	39 (7.2)
Glenoid component revision	13 (19.7)	66 (6.7)	13 (20.3)	64 (11.9)
Humeral component revision	43 (7.8)	553 (56.2)	27 (16.3)	166 (30.8)

a Only the outcome of the 5 most common primary diagnoses have been included.

b Only the outcome of the six most common types of 1st revision have been listed.

c rTSA group head/insert = glenosphere/humeral insert, head only = glenosphere only, cup only = humeral insert only.

c non-rTSA group head/insert = humeral head/glenoid insert, head only = humeral head only, cup only = N/A.

For 1st revision shoulder arthroplasty, the mean age of patients in the rTSA group was 73 years (72 years for males and 73 years for females) and 52% of patients were female. Patients in the non-rTSA group had a mean age of 67 years (65 years for males and 69 years for females) and 60% of patients were female (Table 1).

**Figure 1.**
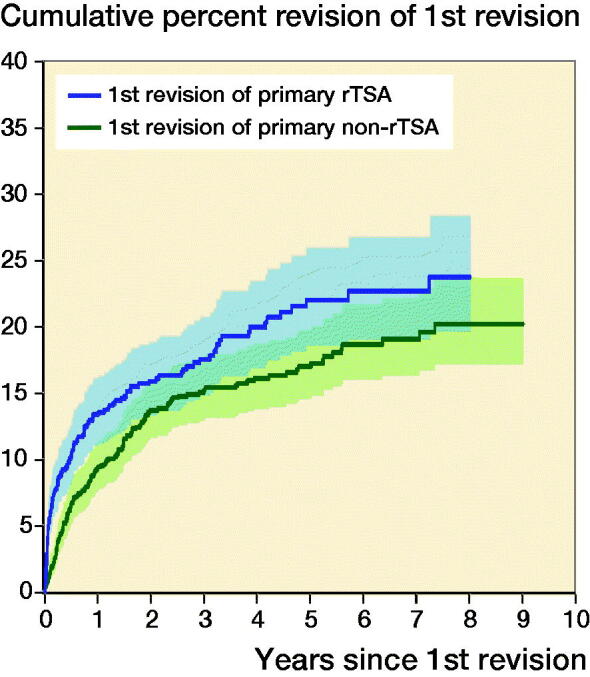
Cumulative percentage of 2nd revision of 1st revision groups rTSA and non-rTSA by type of primary (all diagnoses, excluding 1st revision for infection). HR (CI)—adjusted for age and sex 1st revision of primary rTSA vs 1st revision of primary non-rTSA 0–1 month HR 4.8 (2.5–9.0) 1–6 months HR 1.1 (0.68–1.6) > 6 months HR 1.2 (0.82–1.6)

2nd revisions were undertaken in 18% (n = 129) of the rTSA group and in 16% (n = 157) of the non-rTSA group (Table 1). In the rTSA group, the CPR at 1 year was 13% and at 8 years it was 24%. In the non-rTSA group, the 1-year CPR was 9% and at 8 years it was 20% (Table 2). There was a significantly higher rate of 2nd revisions in the rTSA group for the 1st month only (HR 4.8; CI 2.2–8.9) (Figure 1). After 1 month, there was no significant difference in the risk of a 2nd revision.

**Table 2.** Yearly cumulative percentage of second revision (CPR) of first revision groups rTSA and non-rTSA (all diagnoses, excluding 1st revision for infection)

CPR (CI) after years	Non-rTSA group	rTSA group
1	9.4 (7.7–11)	13 (11–16)
2	14 (12–16)	16 (13–19)
3	15 (13–18)	18 (15–21)
4	16 (14–19)	20 (17–24)
5	17 (14–20)	22 (19–26)
6	19 (16–22)	23 (19–27)
7	19 (16–22)	23 (19–27)
8	20 (17–24)	24 (20–28)
9	20 (17–24)	–

**Figure 2.**
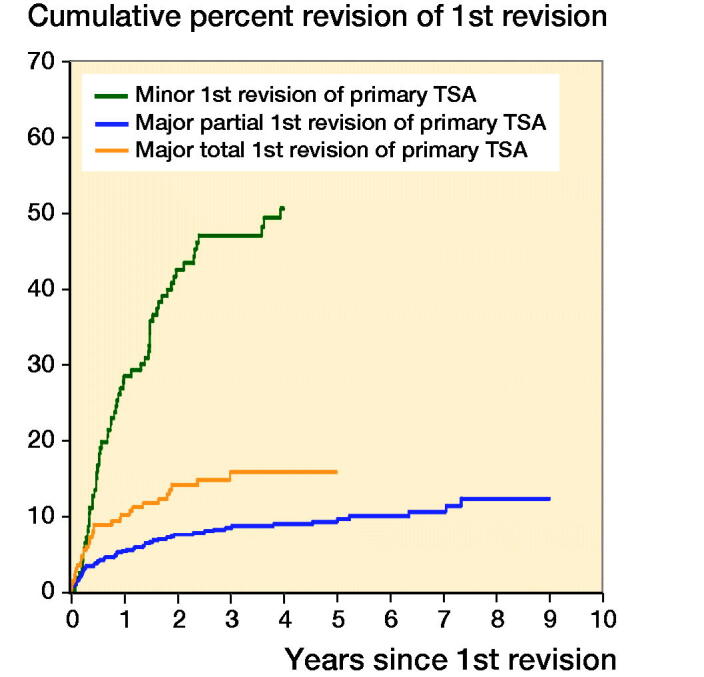
Cumulative percentage of second revision of first revision group non-rTSA by class of the first revision (all diagnoses, excluding first revision for infection). HR (CI)—adjusted for age and sex Non-rTSA group minor revision vs. non-rTSA group major partial revision 0–3 months HR 2.1 (0.95–4.7) 3 months–2 years HR 9.7 (6.1–15) > 2 years HR 9.7 (4.6–20) Non-rTSA group minor revision vs. non-rTSA group major total revision 0–3 months HR 1.1 (0.50–2.6) 3 months–1.5 years HR 5.3 (3.1–9.0) 1.5 years–2 years HR 4.8 (1.8–13) > 2 years HR 5.2 (2.4–12), Non-rTSA group major total revision vs. non-rTSA group major partial revision Entire period HR 1.9 (1.2–2.9)

Analysis of the class of revision found no significant difference in the risk of a 2nd revision for the rTSA group. However, for the non-rTSA group, there was an increased risk of a 2nd revision after 3 months for the minor class compared with other classes of revision (p < 0.001) (Figure 1). The non-rTSA group also had an increased risk of a 2nd revision for total major compared with partial major classes of revision (HR 1.9; CI 1.2–2.9) (Figure 2).

**Figure 3.**
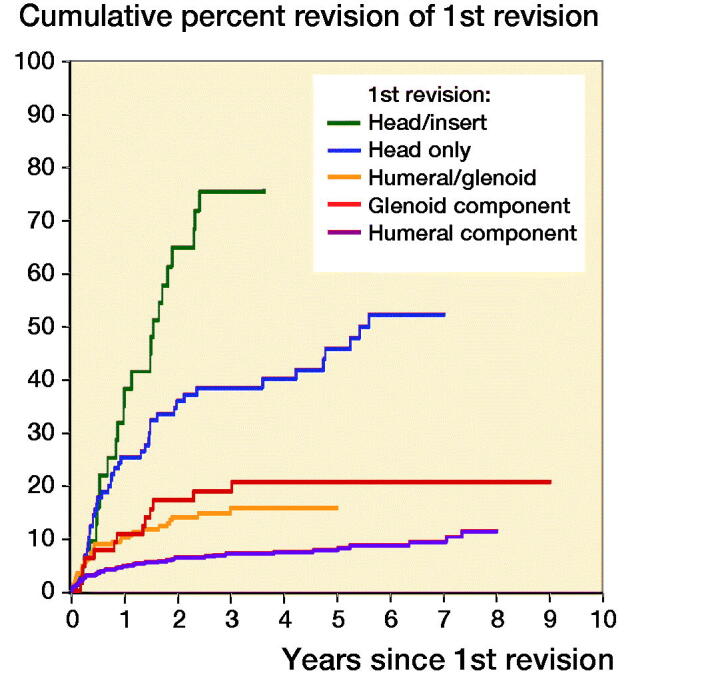
Cumulative percentage of second revision of first revision group non-rTSA by type of revision (all diagnoses, excluding revision for infection). HR (CI)—adjusted for age and sex Humeral head/glenoid insert revision vs. humeral component revision 0–3 months HR = 3.2 (0.74–13) 3 months–1.5 years HR = 15 (7.7–29) > 1.5 years HR = 34 (16–72) Humeral head only revision vs. humeral component revision Entire period HR = 6.8 (4.4–10) Humeral/glenoid revision vs. humeral component revision Entire period HR = 2.2 (1.4–3.4) Glenoid component revision vs. humeral component revision Entire period HR = 2.5 (1.3–4.6)

The type of revision was a statistically significant risk of 2nd revision of cup only compared with head only for the rTSA group (entire period: HR 2.2; CI 1.2–4.2). Other types of revision were not 2nd revision risks. However, humeral head/glenoid insert (after 3 months), humeral head only, glenoid, and glenoid/humeral revision were risk factors for 2nd revision compared with humeral component revision for the non-rTSA group (Figure 3).

The distribution of patients from the rTSA group and the non-rTSA group at 2nd revision by 4 age categories is recorded in Table 1. There was an increased risk of a 2nd revision for patients aged 65–74 years in the rTSA group compared with the non-rTSA group (HR 1.5; CI 1.0–2.1). Patients aged 55–64 years had an increased risk of a 2nd revision in the first 2 weeks in the rTSA group compared with the non-rTSA group (HR 16; CI 2–134), with no significant difference after this time. There was no statistically significant difference in the risk of 2nd revision in any other age group category over the entire period of the study.

The most common diagnosis of primary shoulder arthroplasty that underwent aseptic 1st revision was osteoarthritis, with 54 of 281 in the rTSA group and 142 of 923 in the non-rTSA group undergoing a 2nd revision (Table 1). Primary shoulder arthroplasty undertaken for osteoarthritis had a higher risk of a 2nd revision in the rTSA group compared with the non-rTSA group (HR 1.5; 1.1–2.1). There was insufficient data to determine whether the diagnoses of fracture, osteonecrosis, rheumatoid arthritis, and rotator cuff arthropathy were risk factors for a 2nd revision.

The 3 most common reasons for 2nd revision were similar for both groups of 1st revision shoulder arthroplasty, but the percentages differed. The most common reason for 2nd revision was instability/dislocation and was highest for the rTSA group (54%) compared with the non-rTSA group (36%). There were equal numbers of revisions for loosening (16%) and infection (16%) for the rTSA group. For the non-rTSA group, 2nd revision for loosening was higher (20%) but for infection it was lower (12%) compared with the rTSA group. Collectively, the diagnoses of instability/dislocation, loosening, and infection accounted for 85% of 2nd revision procedures in the rTSA group, and 68% in the non-rTSA group (Table 3). During the 1st month after revision the most common reason for a 2nd revision for both the rTSA group (80%) and the non-rTSA group (62%) was instability/dislocation.

**Table 3.** 2nd revision diagnosis of first revision groups rTSA and non-rTSA (all diagnoses excluding 1st revision for infection)

2nd revision diagnosis	Non-rTSA group		rTSA group	
	2nd rev.	% of 1st	2nd rev.	% of 1st
	n = 157	revisions	n = 129	revisions
	n (%)	n = 991	n (%)	n = 700
Instability/dislocation	57 (36.3)	5.8	70 (54.3)	10.0
Loosening	31 (19.7)	3.1	20 (15.5)	2.9
Infection	18 (11.5)	1.8	20 (15.5)	2.9
Rotator cuff insufficiency	14 (8.9)	1.4	1 (0.8)	0.1
Fracture	4 (2.5)	0.4	6 (4.7)	0.9
Implant breakage glenoid insert	6 (3.8)	0.6	1 (0.8)	0.1
Pain	5 (3.2)	0.5	1 (0.8)	0.1
Dissociation	4 (2.5)	0.4		
Implant breakage glenoid	2 (1.3)	0.2		
Malposition	2 (1.3)	0.2	1 (0.8)	0.1
Metal related pathology	2 (1.3)	0.2		
Arthrofibrosis			1 (0.8)	0.1
Implant breakage humeral	1 (0.6)	0.1	1 (0.8)	0.1
Wear glenoid	1 (0.6)	0.1		
Wear glenoid insert	1 (0.6)	0.1		
Wear humeral cup	1 (0.6)	0.1	1 (0.8)	0.1
Other	8 (5.1)	0.8	6 (4.7)	0.9

## Discussion

The AOANJRR (2018) reported the CPR of primary total shoulder arthroplasty. By comparison, this study found the CPR of first revisions of primary rTSA and non-rTSA to be 2 to 3 times higher at 12 months, and then at year 8 greater than 20%. We are not aware of any large patient study that previously indicated such an increase in CPR for revision shoulder arthroplasty. Others have also reported the outcome of primary TSA and rTSA from the perspective of survivorship (Boileau et al. 2006, Singh et al. 2011, Craig et al. 2019). This study has indicated that, for both rTSA and non-rTSA, the survivorship of the primary was substantially higher than their comparable first revisions (AOANJRR 2018) despite our exclusion of septic 1st revisions. We suggest that studies such as ours serve as a baseline that future revision methods look to and improve upon.

Craig et al. (2019) examined revisions and reoperations in a population-based study of 58,054 primary shoulder arthroplasty procedures and reported similar demographics, but also a high number of non-implant reoperations for primary shoulder arthroplasty. Our study highlights the high 2nd revision rate of shoulder arthroplasty looking exclusively at implant exchange. Both studies confirm the increased operative burden that occurs with shoulder arthroplasty.

There was a statistically significant increase in risk of second revision in the 1st month after the revised primary rTSA over non-rTSA. Our study showed the most common reason for 2nd revision in that period was dislocation/instability. We did not identify specific early (under 1 month) risk factors of second revision on comparing the 2 cohort groups by class or type of revision. It has been previously reported that revision of rTSA is difficult, with a complication rate ranging from 11% to 36% (Sajadi et al. 2010, Austin et al. 2011, Kelly et al. 2012, Patel et al. 2012, Wagner et al. 2018). We confirm that, and the temporal nature of those difficulties. It is likely that the high conversion to reverse shoulder arthroplasty of primary non-rTSA led to the similar CPR results overall after 1 month in this study for the 2 cohort groups.

Our study indicates there would not be confidence in undertaking minor procedures of first revision non-rTSA. Dines et al. (2006) reviewed the outcome of 78 shoulders that underwent revision. They reported component revision was superior to soft tissue revision surgery on the basis of clinical outcome. Their study concluded that glenoid revision had a lower rate of revision compared with other revision procedures. Our results suggest that humeral cup revision has an increased 2nd revision risk. Whilst this may be confounded by diagnosis, there are other minor class revisions that are not 2nd revision risks, such as head revision. Given instability is a common reason for primary rTSA revision, we believe our study is consistent with the findings of Cheung et al. (2018). Cil et al. (2009) reviewed the outcome of 38 humeral revisions for aseptic loosening for total shoulder arthroplasty and hemiarthroplasty. This type of revision surgery gave reliable pain relief but at a high risk of intraoperative complication, with 89% survivorship achieved at 10 years.

There were no clear advantages between differing classes of 1st revision rTSA in our study. Boileau et al. (2013) reported 37 patients who underwent revision rTSA with 11 patients requiring 2nd revision surgery. Overall, 32 retained an rTSA at follow-up at 34 months. Black et al. (2015) reported on 16 patients who underwent component revision after rTSA with a mean follow-up at 5 years. The study reported 6 of the 16 patients underwent further surgery and 9 sustained major complications. There was an improvement in pain and functional scores in their series.

Little is known about the 2nd revision outcome of shoulder arthroplasty. Zumstein et al. (2011) reported a 2nd revision rate of 16% for reverse replacements in a meta-analysis. In the study by Black et al. (2015), of the 6 rTSA revised for instability 2 subsequently required resection arthroplasty and of the 7 revised for glenoid baseplate failure 1 underwent resection arthroplasty. Our rates of 2nd revision were consistent with the combined series by Zumstein et al. (2011).

The diagnosis of osteoarthritis (when controlling for age and sex) was a risk factor for a 2nd revision of shoulder arthroplasty in our study. Singh et al. (2011) examined date of revision for total shoulder arthroplasty for 2,588 shoulders from 1976 to 2008. They found that male sex and rotator cuff disease were independent risk factors for revision of total shoulder arthroplasty. We were only able to confirm younger age as a risk factor for a 2nd revision in the first 2 weeks after the primary shoulder arthroplasty was revised.

The indications for 2nd revisions of rTSA and non-rTSA reflect similar problems to those that occur with primary TSA and primary rTSA. Kelly et al. (2012) observed for reverse total shoulder failed arthroplasty that the indications for the procedure included cuff deficiency, glenoid bone loss, humeral bone loss, or instability of the primary arthroplasty. The Norwegian Registry study of rTSA revisions found infection and loosening to be the most common diagnoses at revision surgery (Fevang et al. 2009). Khan et al. (2009) reported that rotator cuff tears, especially in those with rheumatoid arthritis, were the most common reason for cemented total shoulder arthroplasty revisions.

There are limitations to this study. The differing methods of enrolment and reporting of statistical outcomes of joint registries limit the ability of comparison. The recorded diagnosis is based on a categorical hierarchy and only the primary diagnosis has been used in this study so there may be additional reasons for the revision contributing to revision. This study excluded all primary rTSA and non-rTSA revised for infection. The aseptic study population gave guidance regarding relative implant performance of the 2 cohort groups. Infection of primary shoulder arthroplasty may lead to more complex revisions, a staged response with an outcome more related to the infection than the device. To ensure capture of all revision endpoints, 2nd revision for infection was included. The exclusion of 1st revision for infection, and those 1st revisions subsequently diagnosed as infective managed by antibiotic suppression, may confound infection as reason for 2nd revision. We examined survivorship outcomes measured on a population basis without seeking to select out variations due to implant type, patient selection, surgeons, or geography. This does not represent, necessarily, the optimum achievable results for any particular implant or procedure. This registry data did not include clinical recording such as VAS scores, strength, or range of motion. Similar survivorship may not necessarily imply similar outcomes for pain and function.

## Conclusions

A revised total shoulder arthroplasty has a greater than 20% risk of 2nd revision at 8 years. The type of primary was not a risk factor for a 2nd revision, except in the 1st month, where an rTSA was an increased risk for a 2nd revision compared with a non-rTSA. The risk of a 2nd revision was affected by type and class of the revision, patient age, and the primary diagnosis osteoarthritis when adjusted for sex. The most common reasons for undertaking a 2nd revision were instability/dislocation, loosening, and infection.
